# 50 eV O^+^ ion induced deposition of tin dioxide using tetramethyltin

**DOI:** 10.1016/j.heliyon.2025.e42442

**Published:** 2025-02-04

**Authors:** Satoru Yoshimura, Takae Takeuchi, Masato Kiuchi

**Affiliations:** a Division of Materials and Manufacturing Science, Graduate School of Engineering, Osaka University, Suita, Osaka, 565-0871, Japan; b Cerast Laboratory, Setagaya, Tokyo, 154-0011, Japan

**Keywords:** Tin dioxide, Tin ion beam, Oxygen ion beam, Ion induced deposition, Tetramethyltin

## Abstract

The aim of the present study was to deposit tin oxide films on Si wafers or quartz crystal microbalance plates acting as substrates, using low-energy ion beams. The substrates were held at ambient temperature and two different methods were employed. In one case, the substrates were exposed to a 100 eV beam of Sn^+^ ions together with a flow of gaseous oxygen, and these conditions were found to generate films. Analyses by X-ray photoelectron spectroscopy (XPS) confirmed that these films were made of metallic tin. In other trials, the substrates were exposed to tetramethyltin in conjunction with a 50 eV O^+^ ion beam. This procedure deposited films with a thickness of 30 nm and assessments by XPS and X-ray diffraction established that these films were made of tin dioxide.

## Highlights

• Low-energy ion beam deposition is a powerful technique for the film formation.
• Tin oxide film has attracted much attention because of its various practical applications.
• We used tetramethyltin (TMT) as a source material for tin oxide film formation.
• Tin dioxide films were formed when 50 eV O^+^ ions were injected in conjunction with TMT.

## 1 Introduction

Films composed of materials such as iron [1], carbon [2], silicon carbide [[3], [4], [5]] and silicon oxide [6,7] can be readily formed by exposing various substrates to low-energy ion beams.

Tin oxide films are chemically stable, efficiently transmit visible light and exhibit significant electrical conductivity. Consequently, these films have applications in solar cells [8], gas sensors [9] and touch screens [10]. A number of different processes can be used to generate tin oxide films, including evaporation [11,12], sol-gel techniques [13], chemical vapor deposition (CVD) [14], plasma-enhanced CVD [15] and spray pyrolysis [16,17]. Even so, to date, the use of low-energy ion beams to synthesize films of tin oxide has not been reported.

The present work generated tin oxide films by exposing substrates to a source gas in conjunction with the injection of an ion beam. Tetramethyltin (TMT, Sn(CH_3_)_4_)) was employed as the Sn source in these trials, as this compound has been found to be applicable to the formation of Sn-based films [[18], [19], [20], [21], [22], [23], [24], [25], [26], [27], [28]]. In one set of experiments, the substrates were exposed to Sn^+^ ions with the simultaneous application of gaseous oxygen. Preliminary studies established that substrate exposure to Sn^+^ ions under a high vacuum in the absence of oxygen generated metallic tin films [29]. It was anticipated that the oxygen molecules involved in this process would be dissociated by the Sn ^+^ ions to instead produce tin oxide. Previous work by Kiuchi et al. [30] showed that a film of titanium oxide could be produced by imparting Ti^+^ ions to a substrate in conjunction with exposure to gaseous oxygen, although this phenomenon has not yet been reported with a Sn^+^ ion beam.

In a second set of experiments, the substrate was exposed to a spray of TMT together with an O^+^ ion beam. The expectation was that the Sn-C bonds in the TMT would be ruptured by interactions with the O^+^ ions after which the Sn atoms would be oxidized. Recent work by the present authors established that it was possible to generate films of silicon dioxide by exposing substrates to methylsilane [31], hexamethyldisilane [32], tetraethyl orthosilicate [33] or hexamethyldisiloxane [34] with the simultaneous injection of an O^+^ ion beam. Previous research also confirmed the synthesis of germanium dioxide films using hexamethyldigermane together with an O^+^ ion beam [35]. However, the combination of TMT and O^+^ ions has not yet been evaluated so far.

## 2 Materials and method

**Fig. 1:**
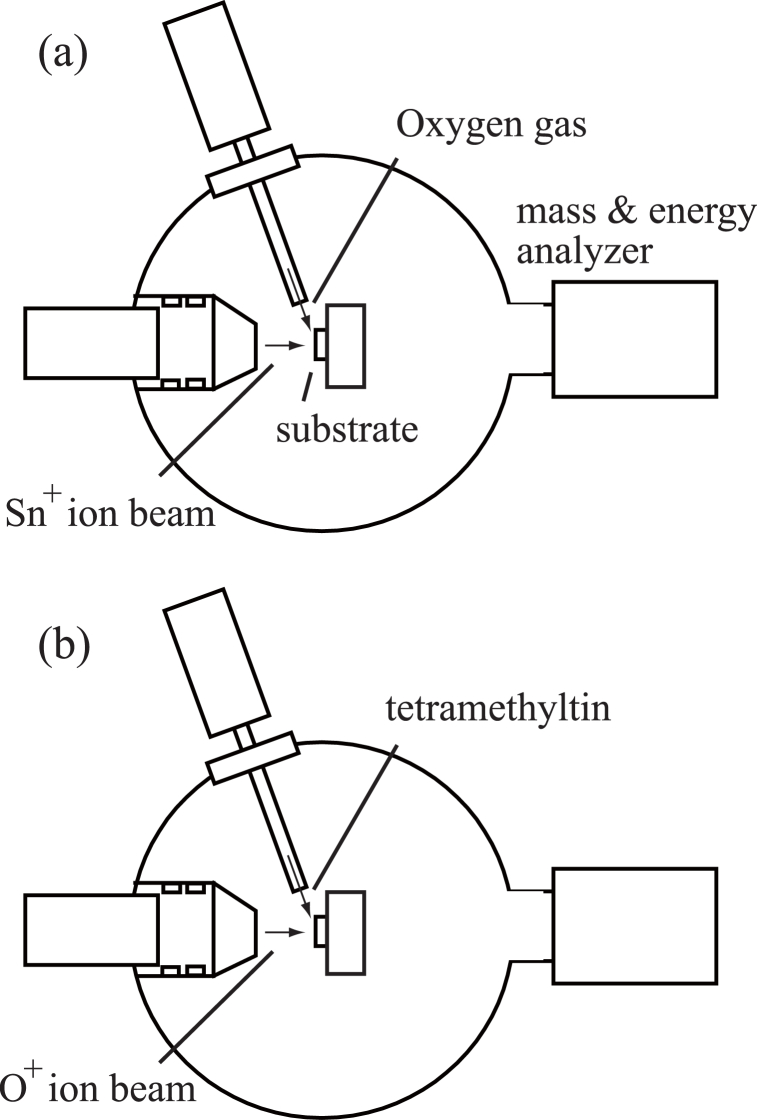
Schematic drawings of the film deposition chamber of the ion beam system. (a) Sn^+^ ion beams were injected to a substrate in conjunction with oxygen gas. (b) O^+^ ion beams were injected to a substrate in conjunction with tetramethyltin.

An ULVAC ion beam system was employed in this study, as described in detail in a prior publication [36]. This apparatus comprised a Freeman-type ion source together with a mass selector and a chamber for film deposition held at a base pressure of 1 × 10^−6^ Pa. This research involved two separate procedures and the film deposition chamber setups for both are presented in Fig. 1(a) and (b), respectively. In each case, either a Si wafer or a quartz crystal microbalance (QCM) plate acting as the substrate was positioned in the chamber at room temperature. The QCM system employed in this work has been described in a previous publication [37]. The mass of the film during and after deposition was ascertained using an ULVAC CRTM-9000 instrument. In procedure (a), gaseous oxygen was imparted to the substrate at a flow rate of 0.5 sccm together with exposure to Sn^+^ ions, as illustrated in Fig. 1(a). During this process, the film deposition chamber was held at a pressure of approximately 1 × 10^−3^ Pa. In procedure (b), the substrate was exposed to TMT by bubbling Ar gas through a stainless steel container holding a quantity of this compound. The flow rate of the gaseous Ar carrying the TMT vapor to the substrate was 0.5 sccm. In these trials, both TMT and O^+^ ions were imparted to the substrate, as illustrated in Fig. 1(b), and the chamber was again held at approximately 1 × 10^−3^ Pa. The films generated using both processes were assessed by X-ray photoelectron spectroscopy (XPS) and X-ray diffraction (XRD)

## 3 Experimental results and discussion

As noted, TMT was used as the source of Sn^+^ ions in the ion source and was obtained by bubbling Ar gas through a stainless steel container filled with this compound. The resulting mixture of Ar and TMT was fed to the ion source, where the TMT was dissociated to generate H^+^, CH_2_^+^ and Sn ^+^ as the primary ions [29]. ^120^Sn is the most abundant Sn isotope and so ^120^Sn ^+^ ions were employed in the present study.

CO_2_ was also fed into the ion source as a precursor for the generation of O^+^ ions. The dissociation of the gaseous CO_2_ was confirmed, along with the production of C^+^, O^+^ and CO^+^ ions. Among these, only the O^+^ ions were obtained from the mass selector.

**Fig. 2:**
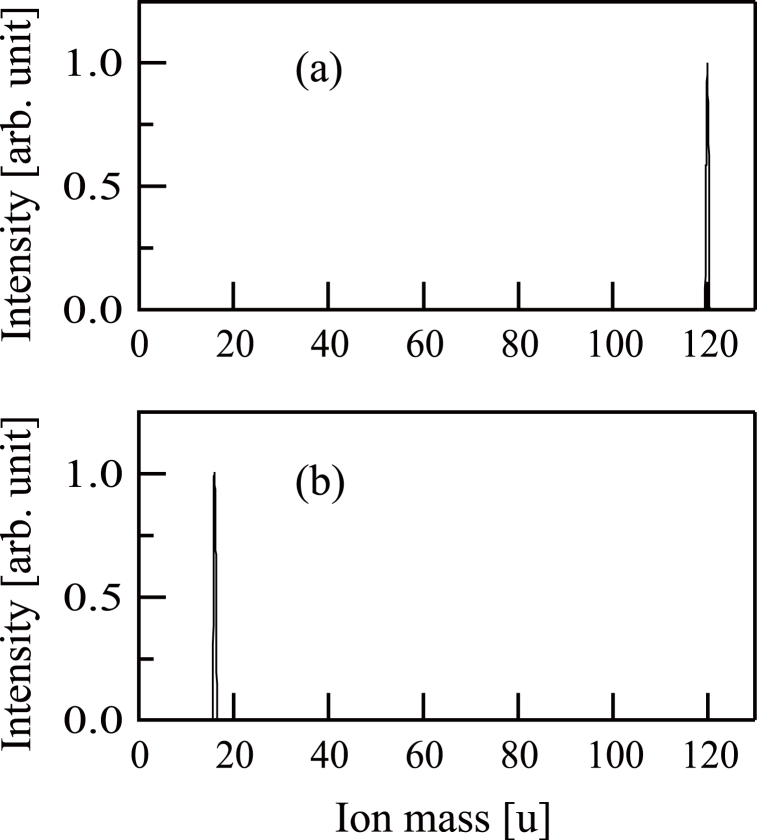
(a) The mass distribution of Sn^+^ ion beams. (b) The mass distribution of O^+^ ion beams.

The mass-based distributions of the ions imparted to the substrate were ascertained in each trial using a Balzers PPM-421 instrument installed in the film deposition chamber [see Fig. 1(a)]. The resulting mass spectra for procedures (a) and (b) are presented in Fig. 2(a) and (b), respectively. Fig. 2(a) exhibits a peak at 120 u, confirming that the beam comprised ^120^Sn ^+^ ions. The mass spectrum presented in Fig. 2(b) shows a peak at 16 u, establishing that O^+^ ions were produced.

**Fig. 3:**
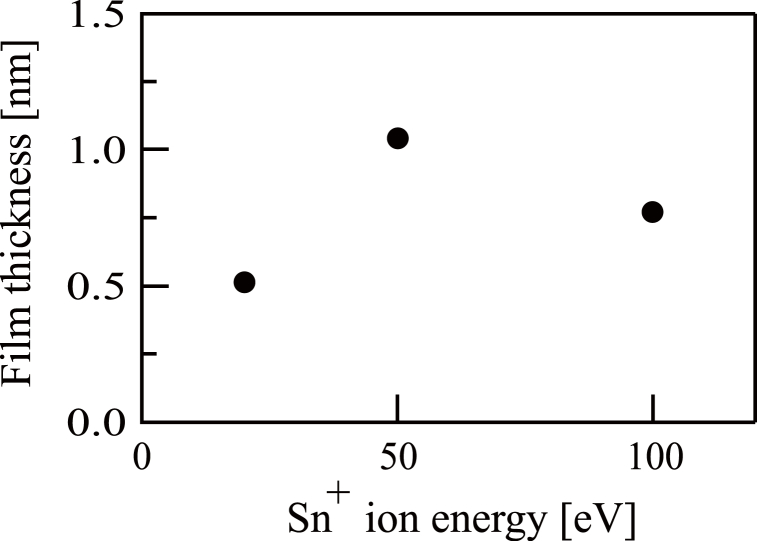
The dependence of film thickness on injecting Sn ^+^ ion energy levels. The film was obtained following the injection of Sn ^+^ ion beams to a quartz crystal microbalance substrate without the supply of oxygen gas.

Prior to the experiments involving either procedure, QCM substrates were irradiated with Sn^+^ ions having energies of 20, 50 or 100 eV for 1 h in the absence of oxygen. A film was found to be formed on the QCM substrate in each of these trials and the mass of each film was determined using the QCM controller. Due to the lack of oxygen, each of these films comprised metallic tin and so the film thicknesses were calculated from the masses assuming a density of 7.3 g/cm^3^. The results are summarized in Fig. 3, which indicates that the 20 eV beam gave the thinnest film. However, because the ion current was maximized at an energy setting of 100 eV, this value was used even though it produced a slightly thinner film when compared with a setting of 50 eV (Fig. 3).

**Fig. 4:**
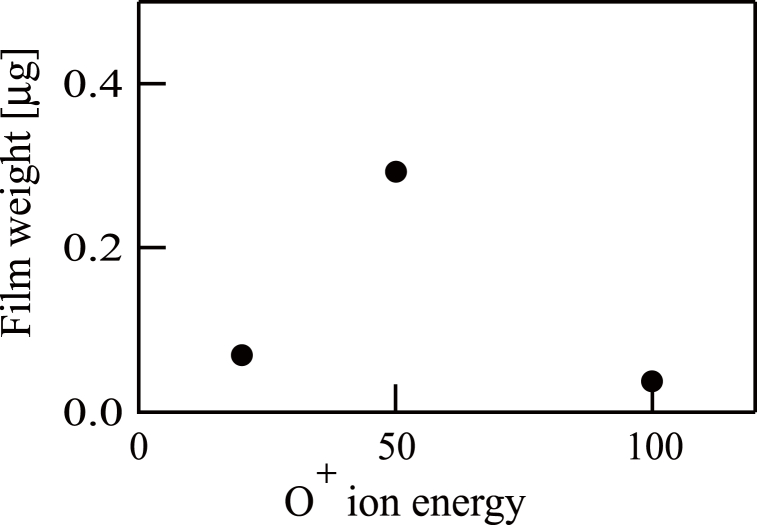
The dependence of deposited film weight on the injecting O^+^ ion energy levels. The film was obtained following the injection of O^+^ ion beams to a quartz crystal microbalance substrate in conjunction with tetramethyltin.

In additional experiments, QCM substrates were exposed to 50 or 100 eV O^+^ ion beams in the absence of TMT, which did not produce films. In addition, substrates were exposed to TMT without an O^+^ ion beam, which again did not generate films. Subsequent to the above, QCM substrates were subjected to contact with TMT with simultaneous irradiation by an O^+^ ion beam having an energy of 20, 50 or 100 eV for 1 h. These experiments did provide films, the masses of which were ascertained using the QCM controller. The data are presented in Fig. 4. It is evident from these data that the 50 eV setting gave the greatest film mass and so this energy value was used for the following trials employing procedure (b).

**Fig. 5:**
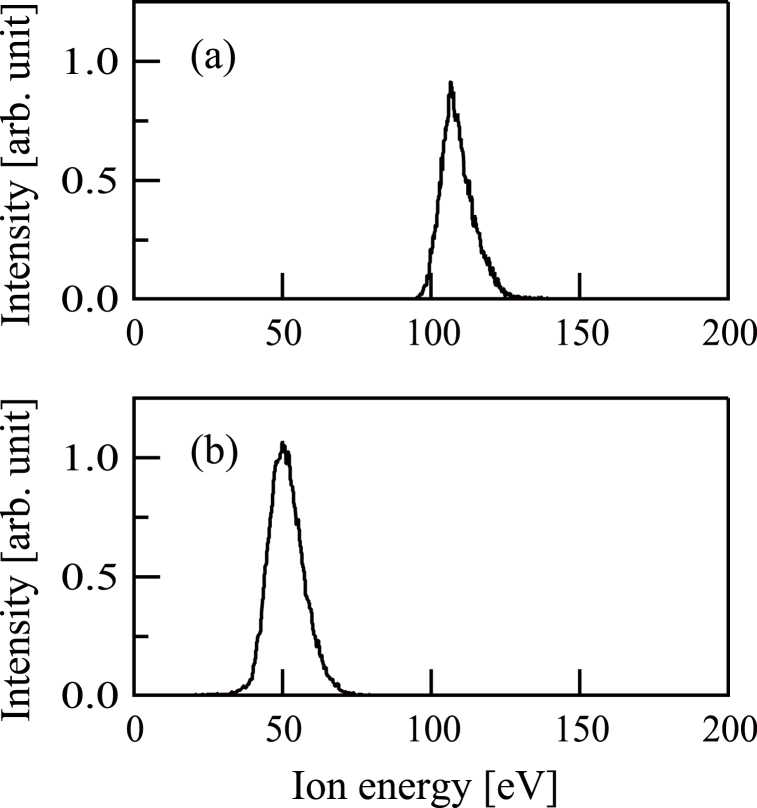
(a) The energy distribution of Sn^+^ ion beams. (b) The energy distribution of O^+^ ion beams.

During the trials involving both procedures, the actual ion energies were determined using the mass and energy analyzer associated with the instrumentation and the Sn^+^ ion energy spectrum is provided in Fig. 5(a). These data show a distribution with an apex at 107 eV that is somewhat higher than the nominal value of 100 eV. The O^+^ ion energy spectrum in Fig. 5(b) indicates that the actual peak value of 50 eV equaled the set value.

During procedure (a), the substrate was treated with gaseous oxygen together with a 100 eV Sn^+^ ion beam having an intensity of 0.3 μA. These experiments had a duration of 6 h. Following each trial, an analysis confirmed that a film had been deposited on the substrate and an assessment using the QCM controller showed a film mass of 2 μg. This film is denoted herein as sample (a). In the case of procedure (b), TMT was introduced into the chamber together with a 50 eV O^+^ ion beam having an intensity of 0.6 μA for 42 h. These conditions provided a 10 μg film with a thickness of 30 nm referred to herein as sample (b).

**Fig. 6:**
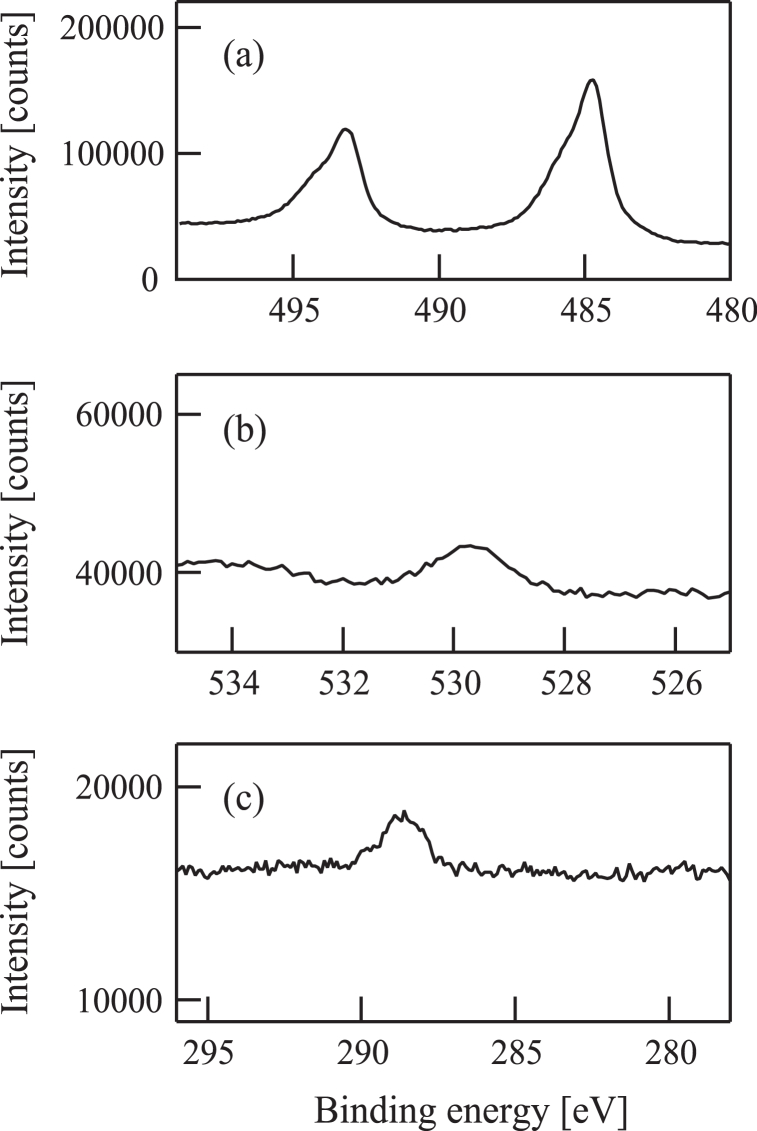
(a) Sn3d, (b) O1s, and (c) C1s X-ray photoelectron spectra of a film deposited on a quartz crystal microbalance substrate by injecting 100 eV Sn^+^ ion beams in conjunction with oxygen gas.

Sample (a) was examined using a Kratos AXIS-165x XPS instrument and the resulting spectra are presented in Fig. 6. The position of the peak in the Sn 3d spectrum in Fig. 6(a) indicates that the film was made of metallic Sn. The O 1s [Fig. 6(b)] and C 1s [Fig. 6(c)] data provide evidence that the film contained low levels of oxygen and carbon.

**Fig. 7:**
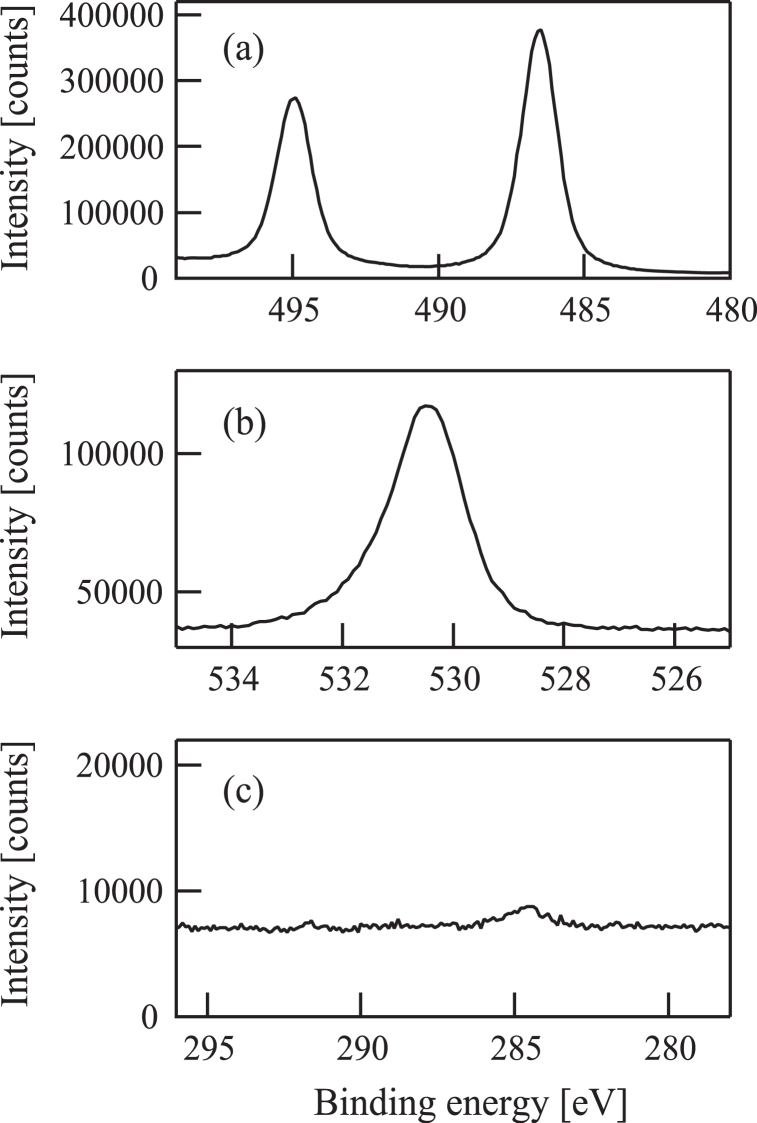
(a) Sn3d, (b) O1s, and (c) C1s X-ray photoelectron spectra of a film deposited on a quartz crystal microbalance substrate by injecting 50 eV O^+^ ion beams in conjunction with tetramethyltin.

Sample (b) was assessed using a PHI Quantera II XPS instrument with the results shown in Fig. 7. From the binding energies associated with the Sn 3d [Fig. 7(a)] and O 1s peaks [Fig. 7(b)] it is evident that the film was made of tin dioxide. The oxygen to tin ratio obtained from these data also provide evidence for this compound, while the C 1s spectrum in Fig. 7(c) indicates that almost no carbon atoms were included in the specimen.

An XRD analysis of the film sample on the QCM substrate was not possible because of the rough nature of the substrate surface. Hence, an additional trial was performed in which a Si wafer was treated with the 100 eV Sn^+^ ion beam and oxygen using similar experimental conditions as applied to produce sample (a). The resulting specimen is referred to herein as sample (a'). In addition, a Si wafer was exposed to the 50 eV O^+^ ion beam together with TMT in the same manner as sample (b) to provide sample (b'). Both these experiments were found to generate films.

**Fig. 8:**
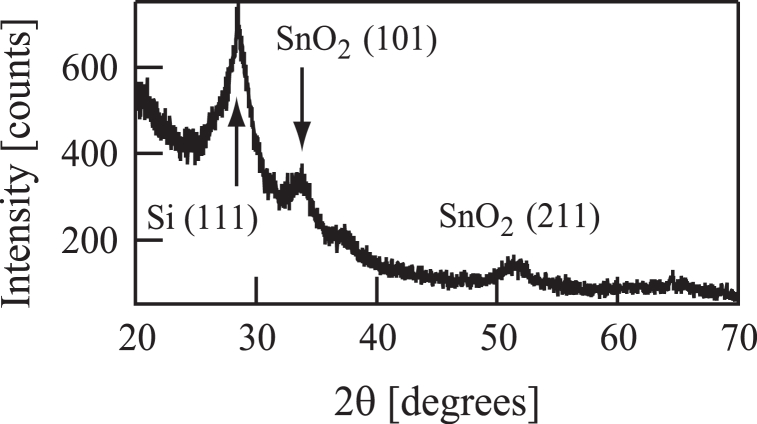
X-ray diffraction pattern (θ-2θ method) of a film deposited on a Si substrate following the injection of 50 eV O^+^ ion beams in conjunction with tetramethyltin.

Sample (a') was assessed by XRD using a RIGAKU RINT2200 diffractometer but did not generate peaks. In contrast, the analysis of sample (b') using a RIGAKU Smart Lab diffractometer with Cu K_α_ radiation produced the pattern shown in Fig. 8. This pattern contains two clear peaks related to the SnO_2_(101) and SnO_2_(211) reflections as well as a Si(111) peak. Hence, this material comprised a crystalline SnO_2_ film.

**Fig. 9:**
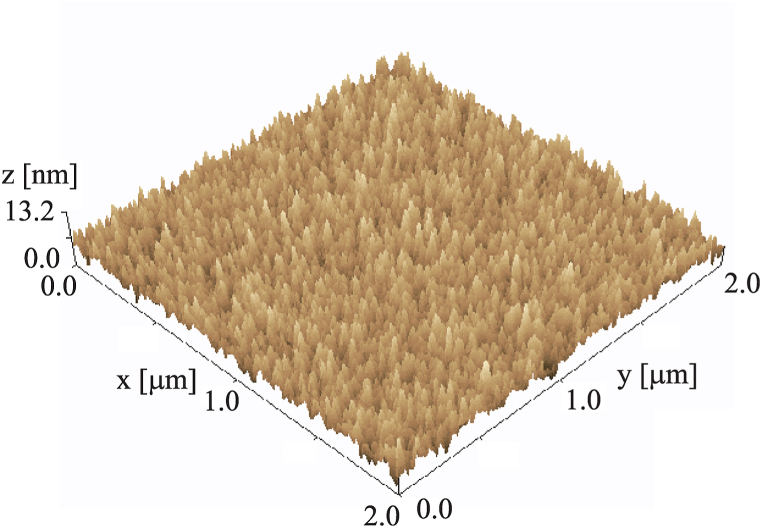
Atomic force microscopy image of a film deposited on a Si substrate following the injection of 50 eV O^+^ ion beams in conjunction with tetramethyltin.

Sample (b’) was then analyzed with atomic force microscopy (AFM) using SPM-9600 (SHIMADZU) instrument. The AFM image is shown in Fig. 9. The root mean square value of surface roughness was 1.6 nm.

In the case of procedure (a), during which the substrate was treated with both Sn^+^ ions and oxygen, the oxygen was expected to dissociate upon reaction with the Sn^+^ ions to produce tin oxide based on prior experimentation with Ti^+^ ions that generated titanium oxide [30]. Interestingly, Fig. 6 demonstrates that sample (a) was made of metallic tin instead of the oxide. From these data it is apparent that Sn reacts differently from Ti under these conditions, presumably due to differences in properties such as oxygen affinity.

Fig. 7(c) confirms that sample (b) contained almost no carbon atoms. During procedure (b), TMT is thought to have initially been adsorbed on the substrate, after which these molecules were dissociated by interactions with O^+^ ions. The Sn-C bond in TMT is much weaker than the C-H bond in the same molecule or the Sn-O bond in SnO_2_ [38] and so the former bond was preferentially cleaved by the O^+^ ions. As a result, Sn and CH_3_ were generated during decomposition of the TMT. The Sn atoms were presumably oxidized by O^+^ ions to generate SnO_2_ on the substrate whereas the CH_3_ was also oxidized to produce volatile species.

Previous studies have examined the deposition of tin oxide films using a number of different techniques but all involved heated substrates [9,[12], [13], [14],^16^,^17^]. For this reason, tin oxide films were rarely obtained based on the poor heat resistance of the substrates. In the present work, however, tin dioxide films were produced using a 50 eV O^+^ ion beam together with TMT and without heating the substrate. The TMT is thought to have been decomposed by interactions with the O^+^ ions, after which the resulting Sn atoms were oxidized by these same O^+^ ions to give tin oxide films at room temperature.

In previous studies with tin oxide films, tintetrachloride (SnCl_4_) was employed as the source material [[15], [16], [17]]. This compound has the potential to damage experimental equipment whereas TMT is relatively safe. Hence, the combination of a 50 eV O^+^ ion beam with TMT as the raw material is a viable approach to growing tin oxide films.

The deposition process for sample (b) requires 42 h, which may be considered impractical for industrial applications. In fact, our ion beam system was prepared for the investigation of ion effects on materials. We have already employed the ion beam system for the measurement of etching yields of materials such as Au [39], polymethyl methacrylate [40], and magnesium oxide [41] by ion injections. The ion current intensity was low in our beam system. In addition, our beam system was not designed to introduce gas in the vacuum chamber. In this paper, a small amount of TMT was introduced to the deposition chamber. For the tin oxide deposition using the method in this paper, it would be necessary to use an ion beam system that can introduce a powerful ion beam and a large amount of TMT into the chamber.

## 4 Conclusion

This study was attempted to use low-energy ion beams to deposit tin oxide films, together with TMT as the source of Sn, and operating all processes at room temperature. In one process, TMT molecules were dissociated by the ion source after which Sn^+^ ions were extracted using the mass selector. Sn^+^ ion beams were then applied to the substrate together with gaseous oxygen, using a beam energy of 100 eV. This process was confirmed to generate a film on the substrate and an XPS analysis showed that the resulting material was metallic tin. In a second procedure, the dissociation of CO_2_ in the ion source provided O^+^ ion beams that were sent to the substrate together with TMT. The energy setting of the O^+^ ion beam during these trials was 50 eV, and a 30 nm thick film was generated. Assessments using XPS and XRD confirmed that this material comprised crystalline tin dioxide. Trials using O^+^ ion beam energies of 20, 50 or 100 eV were carried out and both of the 20 and 100 eV beams provided only thin films. Hence, the optimum energy for O^+^ ions when depositing tin oxide films was determined to be 50 eV.

In conclusion, this study demonstrated that tin dioxide films could be formed using TMT as the Sn source in conjunction with a 50 eV O^+^ ion beam.

## CRediT authorship contribution statement

**Satoru Yoshimura:** Writing – original draft, Project administration, Investigation, Conceptualization. **Takae Takeuchi:** Methodology. **Masato Kiuchi:** Methodology.

## Data availability statement

All relevant data are within the manuscript.

## Additional information

No additional information is available for this paper.

## Funding statement

This research did not receive any specific grant from funding agencies in the public, commercial, or not-for-profit sectors.

## Declaration of competing interest

The authors declare that they have no known competing financial interests or personal relationships that could have appeared to influence the work reported in this paper.
